# Determination of free chlorine based on ion chromatography—application of glycine as a selective scavenger

**DOI:** 10.1007/s00216-020-02885-1

**Published:** 2020-09-18

**Authors:** Mohammad Sajjad Abdighahroudi, Torsten C. Schmidt, Holger V. Lutze

**Affiliations:** 1 Faculty of Chemistry, Instrumental Analytical Chemistry, University of Duisburg-Essen, Universitätsstraße 5, 45141 Essen, Germany; 2 Department of Civil and Environmental Engineering, Institute IWAR, Chair of Environmental Analytics and Pollutants, Technical University of Darmstadt, Franziska-Braun-Straße 7, 64287 Darmstadt, Germany; 3 IWW Water Centre, Moritzstraße 26, 45476 Mülheim an der Ruhr, Germany; 4 Centre for Water and Environmental Research (ZWU), Universitätsstraße 5, 45141 Essen, Germany

**Keywords:** Free available chlorine (FAC), Ion chromatography, Chlorine dioxide, Secondary oxidant, Intrinsic FAC formation

## Abstract

Free available chlorine (FAC) is the most widely used chemical for disinfection and in secondary disinfection; a minimum chlorine residual must be present in the distribution system. FAC can also be formed as an impurity in ClO_2_ production as well as a secondary oxidant in the ClO_2_ application, which has to be monitored. In this study, a new method is developed based on the reaction of FAC with glycine in which the amine group selectively scavenges FAC and the *N*-chloroglycine formed can be measured by ion chromatography with conductivity detector (IC-CD). Utilizing IC for *N*-chloroglycine measurement allows this method to be incorporated into routine monitoring of drinking water anions. For improving the sensitivity, IC was coupled with post-column reaction and UV detection (IC-PCR-UV), which was based on iodide oxidation by *N*-chloroglycine resulting in triiodide. The method performance was quantified by comparison of the results with the *N,N*-diethyl-*p*-phenylenediamine (DPD) method due to the unavailability of an *N*-chloroglycine standard. The *N*-chloroglycine method showed limits of quantification (LOQ) of 24 μg L^−1^ Cl_2_ and 13 μg L^−1^ Cl_2_ for IC-CD and IC-PCR-UV, respectively. These values were lower than those of DPD achieved in this research and in ultrapure water. Measurement of FAC in the drinking water matrix showed comparable robustness and sensitivity with statistically equivalent concentration that translated to recoveries of 102% for IC-CD and 105% for IC-PCR-UV. Repeatability and reproducibility performance were enhanced in the order of DPD, IC-CD, and IC-PCR-UV. Measurement of intrinsic FAC in the ClO_2_ application revealed that the *N*-chloroglycine method performed considerably better in such a system where different oxidant species (ClO_2_, FAC, chlorite, etc.) were present.

**Electronic supplementary material:**

The online version of this article (10.1007/s00216-020-02885-1) contains supplementary material, which is available to authorized users.

## Introduction

Chlorination is in use for disinfection of water since the end of the nineteenth century. Its usage became popular after 1920 and still is the most common form of water treatment worldwide [1]. Free available chlorine (FAC) refers to the amount of chlorine present in water as hypochlorous acid and hypochlorite ions [2]. Because FAC may form chlorinated by-products, chloramination (formation of inorganic monochloramine) as an alternative disinfectant in distribution systems has gained interest [3], which, however, may form nitrosamines [4]. Numerous methods have been developed to measure FAC and chloramine species. Most of the common methods to measure FAC such as iodometric and amperometric titration and reaction with *N,N*-diethyl-*p*-phenylenediamine (DPD) are prone to error, e.g., by buffering capacity, which may aggravate pH adjustment [5] or interferences of several other oxidants such as chloramines and chlorine dioxide (ClO_2_) [5, 6].

FAC can also be formed during ClO_2_ generation [7] as impurity and has to be controlled in ClO_2_ applications. However, most conventional methods cannot selectively measure FAC in the presence of ClO_2_. For example, the DPD method requires two measurements in the presence and absence of scavenger for FAC. FAC can be calculated by subtracting the measurement result for the sample with FAC scavenger from the result for the sample without FAC scavenger. This results in an accumulative error that is especially pronounced in the case of low FAC concentrations in the presence of high ClO_2_ concentrations [8].

Moreover, intrinsic formation of FAC as HOCl in reactions of ClO_2_ with organic compounds has been proposed [9–11]. Recently, this secondary oxidant formation proved to occur in the reaction of ClO_2_ with phenol [12, 13]. Determination of “intrinsic FAC yields” in ClO_2_ application is essential since it affects disinfection, pollutant degradation, as well as by-product formation. Determination of intrinsic FAC yields, e.g., from reactions of ClO_2_, can only be done with a selective scavenger, since formed FAC may also be consumed, e.g., by organic matter.

Glycine is commonly used as the selective scavenger [6] as it reacts fast with FAC [14] and shows a very low reaction rate with ClO_2_ [15]. This provides a selective scavenging step in which the corresponding chloramine is formed. With glycine present in excess over chlorine, the yield of *N*-chloroglycine can be used to assess the FAC concentration. However, due to the lack of an easy and cheap method to measure *N*-chloroglycine, this scavenger was not used for FAC measurement so far.

Formation of *N*-chloroglycine as the product of the reaction between glycine and FAC is known and has been investigated thoroughly [16–18]. Despite the different species of glycine (p*K*a1 = 2.35, p*K*a2 = 9.78) and HOCl (p*K*_a_ = 7.4) [19] present in aqueous solution (cf. speciation of glycine and HOCl Fig. S1 in the Electronic Supplementary Material, ESM), the most relevant reaction according to Armesto et al. [16] is the one shown in Eq. 1.1

The reaction kinetics of Eq. 1 is reported to be between 10^7^ and 10^8^ M^−1^ s^−1^ (*k*_app_ = 10^5^ M^−1^ s^−1^ at pH 7) [14], enabling very good scavenging of FAC. Due to the substitution of hydrogen by chlorine, the p*K*_a_ values of the amino and carboxylic group are lowered. Thus, the anionic species of *N*-chloroglycine is the major species at pH > 4 and can be determined by ion chromatography (IC) (speciation of *N*-chloroglycine cf. Fig. S2, see ESM).

This work aims to develop an alternative method to measure FAC by measurement of *N*-chloroglycine using IC. With the regulation of inorganic anions such as fluoride, nitrite and nitrate, chloride, and sulfate [20–22] and introduction of IC standard methods to quantify them [23, 24], anion-IC became one of the routine analytical methods in drinking water surveillance. Therefore, this method will not only facilitate the measurement of FAC in the presence of ClO_2_ but also be easily incorporated into routine monitoring of drinking water treatment processes. This will allow simultaneous measurement of FAC alongside anions in drinking water facilities with less susceptibility to interferences than conventional methods for FAC determination such as DPD method. The performance of the method will be investigated in FAC measurement in real water matrix, in the presence of ClO_2_, and intrinsic FAC formation. Since no standard of *N*-chloroglycine is commercially available, the performance of the method will be characterized in comparison with DPD as the standard method.

## Material and methods

### Chemicals

All chemicals were commercially available and used as received (purity is presented in parenthesis): Glycine (99%), sodium chlorite (80%), potassium chlorate (99.0%) from Sigma-Aldrich ammonium molybdate tetrahydrate (99%), potassium iodide (99%), and *N,N*-diethyl-*p*-phenylenediamine sulfate (97%) from Alfa Aesar sulfuric acid analytical (95%) from Fisher Scientific, sodium nitrate (99.5%), sodium carbonate (99.5%), and sodium hydrogen carbonate (99%) from Carl Roth and Riedel de Haen, respectively. Na_2_EDTAx2H_2_O (98%), Na_2_HPO_4_x2H_2_O (99.5%), KH_2_PO_4_ (99.5%) from AppliChem. Sodium fluoride (99.5%) from Merck, sodium chloride (99.5%) from Bernd Kraft, sodium bromide (99%) from Fluka, and sodium sulfate anhydrous (99.1%) from VWR chemicals.

Sodium hypochlorite (2 mmol L^−1^) was freshly produced from an 11–15% stock solution (Alfa Aesar) (daily basis). The concentration of OCl^−^ was verified by measuring UV absorption (optical path length 1 cm, pH > 10, ε_OCl_^−^ at 292 nm = 350 M^−1^ cm^−1^ [25]). To take into account that besides HOCl, also Cl_2_ and OCl^−^ may be present in solution, HOCl is referred to as FAC, and its concentration is reported as Cl_2_. *N*-Chloroglycine was produced by mixing the FAC solution with glycine. To avoid the formation of *N,N*-dichloroglycine, FAC to glycine ratio was 0.1 [18]. The concentration of *N*-chloroglycine in the stock solution was confirmed by UV measurements (optical path length 10 cm, pH ≥ 4, ε_*N*-chloroglycine_ at 254 nm = 375 M^−1^ cm^−1^ [26]).

### Instruments

Ultra-pure water was produced using PURELAB ultra from ELGA. For UV-Vis measurement, a Shimadzu UV-1800 spectrometer was used. Ion chromatography was performed using a Metrohm 881 Compact IC pro, including an 814 compact autosampler, 800 Dosino, and a MagIC Net 3.1 software for data acquisition and processing. The IC system was coupled with a post-column reaction (HT reactor Metrohm) using Spetec Perimax peristaltic pump for reagent delivery and a 944 UV detector. For separation, Metrosep A Supp 4, 5, 7, 10, and 16 (all with dimensions of 250 mm × 4.0 mm) columns were tested, and eventually, Metrosep A Supp 7 was used.

### Ion chromatography method

The injection volume was set to 300 μL. Used eluent was a 1.6 mmol L^−1^ sodium carbonate solution delivered with a flow rate of 0.8 mL min^−1^. To increase the sensitivity of the *N*-chloroglycine method, an iodometric post-column reaction combined with UV detection (PCR-UV) was used according to DIN EN ISO 11206 [27]. Organic chloramines such as *N*-chloroglycine react with iodide to form triiodide (Eq. 2) [26]. The triiodide formed in this reaction can be measured by UV absorption at 352 nm with high sensitivity (ε = 26,000 M^−1^ cm^−1^ [26]).

2 $$ RN HCl+3{I}^{-}+{H}^{+}\to RN{H}_2+{Cl}^{-}+{I}_3^{-} $$

PCR included potassium iodide (KI) and a catalyst, delivered by a peristaltic pump with a combined flow rate of 0.2 mL min^−1^. The concentration of the KI solution was 270 mmol L^−1^. The catalyst solution was composed of ammonium molybdate tetrahydrate (50 μmol L^−1^) and sulfuric acid (100 mmol L^−1^). A scheme of the setup is shown in Fig. S3 (see ESM). Scavenging of FAC by glycine was performed by adding 10 mL of sample to 1 mL of a 5 mmol L^−1^ glycine solution (i.e., more than 5 times excess of glycine over the highest expected FAC concentration (70 μmol L^−1^ Cl_2_)). Then the sample was analyzed with IC using a conductivity detector (IC-CD) and post-column reaction with a UV detector (IC-PCR-UV). Using PCR-UV also allows measuring monochloramine in case the cation suppressor is by-passed (see Text S1 in ESM).

### DPD method

The applied DPD method was based on DIN EN ISO 7393-2 [28]. In brief, 0.5 mL of buffer (phosphate, ≈ 500 mmol L^−1^) and 0.5 mL of DPD solution (≈ 4.2 mmol L^−1^) were added to a beaker; afterward, 10 mL of the sample was added. After a reaction time of 1 min, the absorbance at 510 nm was measured with a UV-Vis spectrometer. The buffer solution was prepared by dissolving 30.1 g Na_2_HPO_4_x2H_2_O, 46.0 g KH_2_PO_4_, 0.8 g Na_2_EDTAx2H_2_O in a 1000-mL volumetric flask and filled up with ultrapure water. For the preparation of DPD reagent solution 250 mL ultrapure water, 0.2 g Na_2_EDTAx2H_2_O, and 2 mL concentrated sulfuric acid were added to a 1000-mL volumetric flask. Then, 1.1 g water-free DPD was added to the same volumetric flask. After the dissolution of all reagents, it was filled up to the mark with ultrapure water.

### Measurement of FAC in the presence of ClO_2_

To measure FAC in the presence of ClO_2_, samples were measured with the DPD method [6]. In short, samples with different ratios of FAC and ClO_2_ were divided into two fractions (A and B). Fraction A was measured with the DPD method. The reading of these samples (reading A) represents both ClO_2_ and FAC. Glycine was added to fraction B, which scavenges FAC. Afterward, fraction B was measured by the DPD method, which gives a signal for ClO_2_ (reading B). Subtracting reading B from reading A equals FAC. A scheme of the procedure for the measurement of FAC in the presence of ClO_2_ is presented in Fig. S4 (see ESM). The glycine-containing samples (fraction B) were also measured with IC for the determination of *N*-chloroglycine.

To measure intrinsic FAC formed in ClO_2_ applications, samples containing 10 μmol L^−1^ phenol were spiked with different ClO_2_ concentrations at pH 7 at room temperature (sample A) and was treated as described above for fraction A. This reading represents both the remaining ClO_2_ and intrinsic FAC formed in the sample (note that the reaction of ClO_2_ with phenol yields FAC (see above)). Parallel to these samples, another set of samples in the presence of glycine for scavenging FAC was spiked with ClO_2_ (sample B). In both samples, DPD was added 1 h after the ClO_2_ dosage. Subtraction of the reading of samples B from samples A equals intrinsic FAC. A scheme of the procedure for the measurement of intrinsic FAC is presented in Fig. S5 (see ESM). Furthermore, sample B was measured with IC directly without further treatment for *N*-chloroglycine determination.

### Method validation

Due to the lack of *N*-chloroglycine standards, the FAC samples were always measured with both the *N*-chloroglycine and DPD method, and the results were compared for method validation purposes. The limit of quantification was calculated by a signal to noise ratio of 10 using maximum noise variation close to the corresponding peak. Method standard deviation was calculated by dividing the residual standard deviation of the calibration curve to its slope. Linearity was compared with DPD in the 30–5000 μg L^−1^ Cl_2_ range suggested in the DIN EN ISO 73 93-2 method for colorimetric determination of free chlorine using DPD [28]. Recoveries of IC-CD and IC-PCR-UV were calculated relative to DPD, and repeatability and reproducibility are calculated as the standard deviation of triplicate intraday and interday measurement, respectively. *T* test was used to compare the resulting values from DPD and *N*-chloroglycine methods based on Miller et al. [29]. In that, a pooled estimate of standard deviations is calculated based on Eq. 3 and used to calculate *t* value based on Eq. 4.

3 $$ s=\frac{\left({n}_1-1\right){s}_1^2+\left({n}_2-1\right){s}_2^2}{\left({n}_1+{n}_2-2\right)} $$

4 $$ t=\frac{{\overline{x}}_1-{\overline{x}}_2}{s\left(\frac{1}{n_1}+\frac{1}{n_2}\right)} $$

where *s*_i_ is the standard deviation of the measurements using either IC-CD, IC-PCR-UV, or DPD method, and *s* is the pooled estimate of the standard deviation. *n*_i_ is the number of corresponding measurements for each method and ${\overline{x}}_i$ is the average of the resulting values.

## Results and discussion

### Method development and validation

**Fig. 1:**
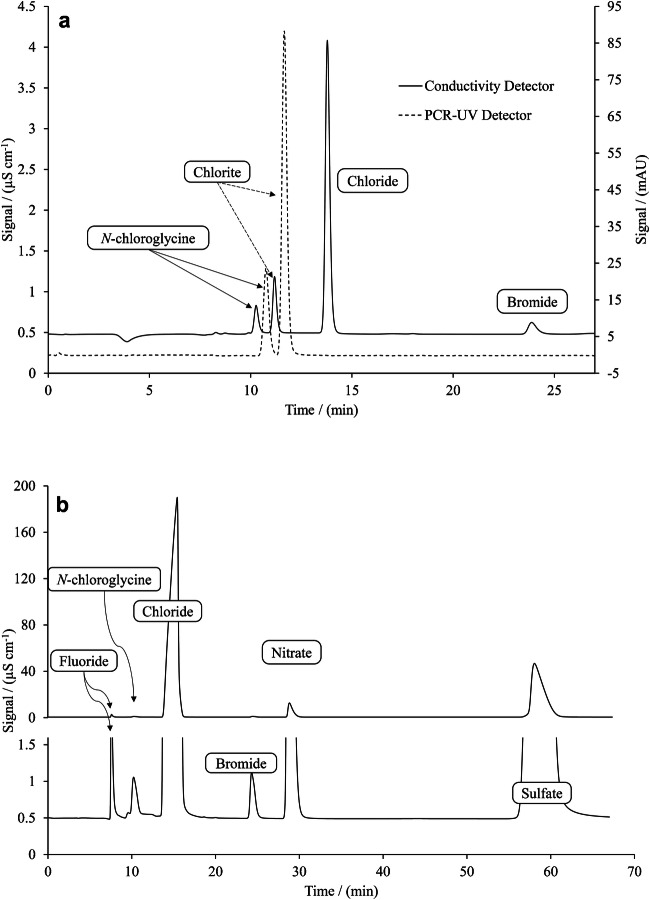
IC chromatograms of *N*-chloroglycine method using eluent 1.6 mmol L^−1^ sodium carbonate, flowrate of 0.8 mL min^−1^, PCR [KI] = 270 mmol L^−1^, [ammonium molybdate tetrahydrate] = 50 μmol L^−1^, [sulfuric acid] = 100 mmol L^−1^, KI was added separately, flowrate of PCR reagents 0.2 mL min^−1^, wavelength of UV detection 352 nm, injection volume 300 μL, (**a**) an ultrapure water sample containing 200 μg L^−1^ FAC detected as *N*-chloroglycine, 200 μg L^−1^ ClO_2_ detected as chlorite resulting from the alkaline decomposition of ClO_2_ in high pH of the eluent, and chloride and bromide as impurities of sodium hypochlorite solution (**b**) a tap water sample spiked with 800 μg L^−1^ FAC and measured with conductivity detector (upper part of the figure shows the total chromatogram, and the lower part represents an enlarged view of the baseline and smaller peaks). Note that chlorite and chlorate were not present in the drinking water sample and thus not detected

In a preliminary set of experiments, different columns, including A Supp 4, 5, 7, 10, and 16, were tested regarding retention behavior. However, no signal for *N*-chloroglycine was detected using A Supp 10 and 16, while in separation using A Supp 4, 5, and 7 columns, a peak was observed. It was noted that the first group uses polystyrene-divinylbenzene copolymer with quaternary ammonium as solid-phase material, while polyvinyl alcohol with quaternary ammonium is the stationary phase for the second group, according to the manufacturer. This can indicate that *N*-chloroglycine might react with the different solid-phase material as a chloramine. Moreover, by using the standard carbonate eluents of A Supp 4, 5, and 7 columns, chlorite and *N*-chloroglycine peaks coeluted to some extent. By utilizing A Supp 7 and a weak eluent with a concentration of 1.6 mmol L^−1^ sodium carbonate with a flow rate of 0.8 mL min^−1^, it was possible to separate these two compounds (Fig. 1a) and also common anions in the drinking water sample (Fig. 1b). It is worth mentioning that for samples that do not contain chlorite, standard or stronger eluents with higher flow rates can be utilized. It is also possible to use A Supp 4 or 5, which have lower column capacity. Employing these recommendations will reduce the retention of compounds such as sulfate and, thus, decrease the overall runtime. Different solid-phase material behavior, as well as different suppression techniques, can also be subject to future research.

To investigate the performance of the *N*-chloroglycine method, several series of samples were measured with DPD and IC. The respective method performance parameters for the *N*-chloroglycine method using both IC-CD and IC-PCR-UV, as well as common anions in drinking water, are shown in Table 1. The employment of the *N*-chloroglycine method is most interesting in the case of ClO_2_ applications (see 3.2) since it allows to measure all main by-products (chlorite, chloride, chlorate, and intrinsic FAC) simultaneously; therefore, the method performance for chlorite and chlorate is also presented in Table 1. Gordon et al. [8] reported LOQ of colorimetric DPD method for FAC to be 10 μg L^−1^ Cl_2_. This is slightly below the LOQ determined in our study as well as DIN EN ISO 7393-2 [28], which can be explained by differences in the experimental setup. Considering that the LODs and LOQs were determined in different laboratories and by different persons, the LOQ reported in literature agrees with the LOQ determined in the present study.

**Table 1:** Method performance parameters for measurement of FAC and drinking water anions using different methods (FAC = added HOCl, expressed as Cl_2_ equivalents)

Method	Slope or sensitivity/(peak area × μg^−1^ L)	Intercept/(peak area)	*R*^2^ correlation coefficient	Method standard deviation^a^/(μg L^−1^)	LOQ^b^/(μg L^−1^)
FAC by IC-CD	(3.8 ± 0.1) × 10^−5^	− 0.0009 ± 0.0009	0.9949	2.6	24
FAC by IC-PCR-UV	(3.39 ± 0.07) × 10^−2^	− 0.08 ± 0.04	0.9989	1.2	13
FAC by DPD	(2.18 ± 0.09) × 10^−4^	0.0095 ± 0.0005	0.9947	2.7	68
Fluoride	(44.2 ± 0.5) × 10^−4^	− 0.016 ± 0.003	0.9995	0.79	2.2
Chloride	(15.6 ± 0.2) × 10^−3^	− 0.06 ± 0.01	0.9992	1	4
Bromide	(9.1 ± 0.1) × 10^−4^	− 0.0015 ± 0.0006	0.9995	0.77	3.9
Nitrate	(12.7 ± 0.3) × 10^−4^	0 ± 0.001	0.9987	1.3	5
Sulfate	(24.8 ± 0.6) × 10^−4^	− 0.007 ± 0.003	0.9993	0.90	12.6
Chlorite by IC-CD	(12.9 ± 0.5) × 10^−4^	− 0.03 ± 0.01	0.9955	8.3	28
Chlorite by IC-PCR-UV	(19 ± 0.4) × 10^−2^	− 2.9 ± 0.9	0.9983	4.9	19
Chlorate	(6.81 ± 0.2) × 10^−4^	− 0.002 ± 0.005	0.9938	10	40

^a^Residual standard deviation/sensitivity (slope)

^b^S/*N* = 10

Moreover, measurement of FAC in a range close to the LOQ shows that the 95% confidence interval for IC-PCR-UV is slightly smaller than for DPD (ESM Fig. S6).

Another important parameter is the linearity of the method. According to DIN EN ISO 7393-2 [28], the DPD method has a range from 30 to 5000 μg L^−1^ Cl_2_ and, thus, both methods are compared in this range. As shown in Fig. S7 (see ESM), the calibration line for *N*-chloroglycine method has a higher correlation coefficient compared with the DPD method. Figure S8 (see ESM) represents the residuals of the DPD and *N*-chloroglycine method calibration lines, and a trend can be seen in the DPD method, indicating a non-linear behavior. Previous results of Gordon et al. [30] using KMnO_4_ to oxidize DPD showed similar behavior. This is due to further oxidation of the Würster dye to form the colorless imine. The lower the ratio of DPD to chlorine, the higher the chance of the imine formation, which may limit the working range of the colorimetric DPD method. Gordon et al. [30] managed to overcome this problem by using 100 times higher concentration of DPD (110 g L^−1^ ≈ 0.451 mol L^−1^) in the DPD method. This problem is not observed for the glycine method, which had a glycine concentration as low as 375 mg L^−1^ (≈ 0.005 mol L^−1^).

**Fig. 2:**
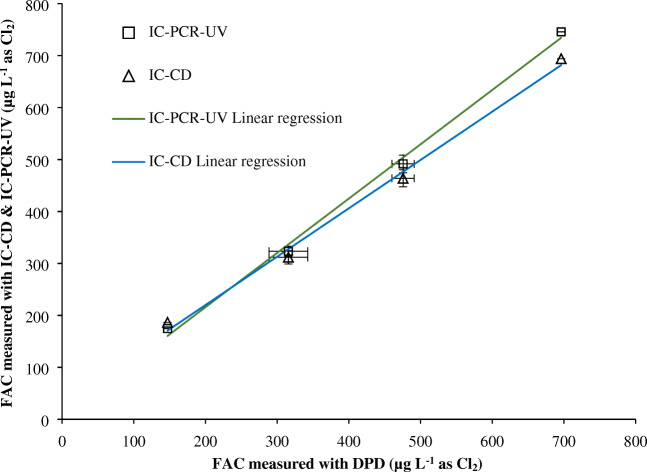
FAC measured by *N*-chloroglycine method using IC-CD and IC-PCR-UV compared with the DPD method. The tap water sample was spiked with different FAC concentrations. Error bars show the standard deviation of triplicate measurements. (FAC = added HOCl, expressed as Cl_2_ equivalents)

Method performance in tap water matrix was investigated by characterizing recovery, repeatability, and reproducibility. To that end, different concentrations of FAC were spiked to tap water at typical drinking water concentrations. Results of IC-CD and IC-PCR-UV detection are compared with those of the DPD method in Fig. 2. The slopes of both lines are close to one, indicating a good correlation between the measurement of IC methods with DPD. Moreover, the concentrations of the triplicate measurements with these methods were statistically equivalent (α = 0.05). The average recoveries of IC-CD and IC-PCR-UV calculated relative to the DPD method were 102 and 105%, respectively. Thus, the determined concentrations in the drinking water matrix are in very good agreement with the concentrations obtained in ultrapure water (Table 1). For other matrices, it is recommended to calculate the individual matrix-dependent LOQ on the basis of the recovery rates in analogy to Mechelke et al. [31]. The relative standard deviation (RSD) of the samples measured during the same day (repeatability/intraday) with DPD (5.9%), IC-CD (3.7%), IC-PCR-UV (3.2%), indicates similar or better performance of IC methods (IC-PCR-UV and IC-CD). Reproducibility (interday) measurements performed within 3 weeks with freshly prepared eluents also showed statistically equivalent concentration (*α* = 0.05) with similar relative standard deviations for IC-CD (2.4%) and IC-PCR-UV (3.4%) compared with DPD (3.9%) (average). As mentioned above, it is possible to measure FAC alongside common anions in drinking water. Concentrations of anions present in the tap water used for the method validation study are presented in Table S1 (see ESM). Chromatograms of IC-CD and IC-PCR-UV corresponding to one of the spiked tap water samples are shown in Fig. 1b.

From the results, it can be concluded that the *N*-chloroglycine method has comparable performance with the DPD method in the measurement of FAC in a water matrix that is common in drinking water. However, DPD oxidation can also happen in reaction with ClO_2_, ozone, H_2_O_2_, permanganate, bromine, iodine [32], disinfection by-products such as inorganic [33, 34], and organic chloramines [35], bromamines [36] and bromochloramines [37], chlorite and chlorate [38], and oxidized manganese species (MnO_2_, MnO_4_^−^) [6]. This exacerbates a reliable measurement of FAC using DPD, while in the new IC-based method, these interferences do not exist due to the measurement of *N*-chloroglycine as the product of the reaction between FAC and glycine.

One point to discuss in *N*-chloroglycine method is the presence of glycine as one of the free amino acids with the highest concentration in raw water [39]. However, the concentration of free amino acids in raw water is reported to be around 2.5 μg L^−1^ at highest with a maximum of 20% glycine content. Moreover, compared with FAC, glycine concentration is way lower, and in excess of FAC, *N,N*-dichloroglycine will form [18, 40, 41]. *N,N*-Dichloroglycine is subject to fast auto-decomposition (*t*_1/2_ = 13.0 min) [42]. All in all, it is implausible that background glycine concentration will cause any interferences due to low abundance and further degradation of *N,N*-dichloroglycine.

### Measurement of FAC in presence of ClO_2_

The performance of the *N-*chloroglycine method for measurement of FAC impurities in ClO_2_ production was compared with the DPD method for the measurement of FAC. As shown in Fig. S9 (see ESM), method performance for DPD and *N*-chloroglycine methods were comparable. Although the slope of the *N*-chloroglycine method was slightly closer to the ideal value (i.e., 1), they were statistically equivalent (*α* = 0.05). It can be concluded that the *N*-chloroglycine method is capable of FAC measurement in the presence of ClO_2_.

Methods that are used to measure intrinsic FAC in ClO_2_ applications so far have some disadvantages. For example, in the ABTS-based method, ammonia is used as the scavenger, and monochloramine concentration is calculated by subtracting ClO_2_ from the total available chlorine fraction [13]. This can affect the accuracy of the ABTS method based on literature [43]. It is also needed to add HgCl_2_ to the sample to complex iodide and prevent the overestimation of ClO_2_ concentration, which is not advocated due to its high toxicity. Moreover, ABTS^•^^+^ (the colored compound subject to measurement) is not stable and reduced in the presence of hydrogen-donating antioxidants [44]. It also can react with basic carbonates and other oxidants [45]. Therefore, this radical is commonly used for the measurement of antioxidant capacity [46, 47].

Additionally, in comparison with glycine, ammonia has a lower reaction rate with FAC [48]. Lower reaction rate might hinder proper scavenging in the presence of compounds like amino acids that have high reaction rates with FAC [14]. This is also true for other selective scavengers like trimethoxybenzene (TMB) [49]. Moreover, monochloramine decomposes faster compared with *N*-chloroglycine [50] and is a stronger oxidant [51] reactive towards moieties such as phenol [52]. Monochloramine is also volatile [53], and for these reasons, glycine might be a more suitable scavenger.

**Fig. 3:**
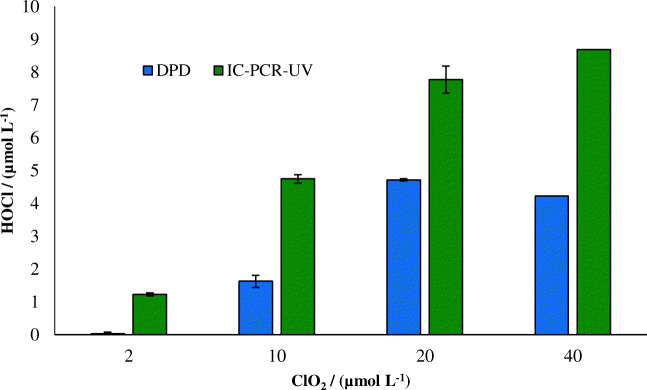
Measurement of intrinsic FAC formation from the reaction of ClO_2_ with phenol using DPD and *N*-chloroglycine (IC-PCR-UV) method. 10 μmol L^−1^ phenol samples were treated with 2–40 μmol L^−1^ ClO_2_. Error bars show the standard deviation of triplicate measurements. Measured at room temperature after 1 h reaction time, pH = 7, [phosphate buffer] = 5 mmol L^−1^

Figure 3 represents the results for the measurement of intrinsic FAC in the ClO_2_ application. Wajon et al. [9] suggested a reaction mechanism for phenol and ClO_2_ with a 50% intrinsic FAC yield. This mechanism has lately been proven by Terhalle et al. [12] and Rougé et al. [13]. The results for the intrinsic FAC measurement based on the DPD method were lower than those obtained by the *N*-chloroglycine method, showing a considerably better performance for the *N*-chloroglycine method. Overall, quantification of intrinsic FAC yields with DPD is very difficult (if not entirely unfeasible). Time-resolved measurement of intrinsic FAC is not possible with any method that needs subtraction of ClO_2_ from ClO_2_ + FAC, including DPD.

### Stability of reagents of the *N*-chloroglycine method

Glycine stock solution in ultra-pure water can be kept for months without any relevant change (note that biodegradation has to be ruled out), while DPD solutions can be oxidized by oxygen [54]. This phenomenon is a lot faster at higher pH; thus, the DPD reagents always need to be kept in acidic conditions [54]. Therefore, the *N*-chloroglycine method is very robust compared with DPD concerning the chemical storage of the reagents.

*N*-chloroglycine, on the other hand, can undergo auto decomposition. Table 2 shows the decomposition rate of *N*-chloroglycine measured by the UV spectrometer at 254 nm. The results confirm the lower stability of *N*-chloroglycine at pH 4 (acid-catalyzed disproportionation [55]). This fact and the run time of such a method can be regarded as a disadvantage compared with methods such as DPD that provide the results within minutes. However, instability of *N*-chloroglycine can lead to around 8% loss at pH 7 in 8 h (ESM Fig. S10). On the other hand, a study on the color fading of the DPD method showed a 3% up to 29% decrease between 1 and 5 min after mixing of DPD and FAC [54]. Further information on the decomposition of *N*-chloroglycine is presented in Text S2 (see ESM).

**Table 2:** Decomposition rate and half-life time of *N*-chloroglycine at different pH values measured by UV absorption at 254 nm (optical path length = 10 cm, [phosphate buffer] = 5 mmol L^−1^)

	pH 4	pH 7	pH 10
*k* (s^−1^)	8.96 × 10^−6^	2.83 × 10^−6^	3.53 × 10^−6^
t_1/2_ (hour)	21.5	68.0	54.5

## Conclusion

The *N*-chloroglycine method allows determining FAC and other ions simultaneously with good sensitivity. This allows the incorporation of FAC measurements into standard IC methods to determine other anions that have to be monitored in drinking water. Measurement of FAC alongside other anions may be especially important for applications of ClO_2_ where chlorite and intrinsic FAC have to be monitored. Reagents of the *N*-chloroglycine method and *N*-chloroglycine itself are fairly stable. The *N*-chloroglycine method may also be a suitable reference method to corroborate FAC measurements by other methods such as the DPD method. The simplicity of the scavenging procedure alongside the stability of glycine and *N*-chloroglycine makes this method very robust and reliable. In the case of FAC measurements in the presence of other oxidants, the selectivity of *N*-chloroglycine formation for FAC alongside the chromatographic separation of the compound provides an automated method with low potential of interferences.

## Electronic supplementary material

ESM 1 (PDF 710 kb)
