# Supplementary material for: Determination of free chlorine based on ion chromatography—application of glycine as a selective scavenger
Source: Anal Bioanal Chem. 2020 Sep 18;412(28):7713–22. doi: 10.1007/s00216-020-02885-1 (PMC7550385; doi:10.1007/s00216-020-02885-1)
Supplement: Supplementary file 1 — (PDF 710 kb) [file 216_2020_2885_MOESM1_ESM.pdf]

Electronic Supplementary Material

Determination of free chlorine based on ion chromatography - application of glycine as a selective scavenger

Mohammad Sajjad Abdighahroudi[1,2], Torsten C. Schmidt[1,3,4] and Holger V. Lutze[1,2,3,4]

[1] University of Duisburg-Essen, Faculty of Chemistry, Instrumental Analytical Chemistry, Universitätsstraße 5, D-45141 Essen, Germany

[2] Technical University of Darmstadt, Department of Civil and Environmental Engineering, Institute IWAR, Franziska-Braun-Straße 7, D-64287 Darmstadt, Germany

[3] IWW Water Centre, Moritzstraße 26, D-45476 Mülheim an der Ruhr, Germany

[4] Centre for Water and Environmental Research (ZWU), Universitätsstraße 5, D-45141 Essen, Germany

Corresponding author: Tel.: +49 6151 16-20459; fax: +49 6151 16-20305; e-mail: h.lutze@iwar.tu-darmstadt.de

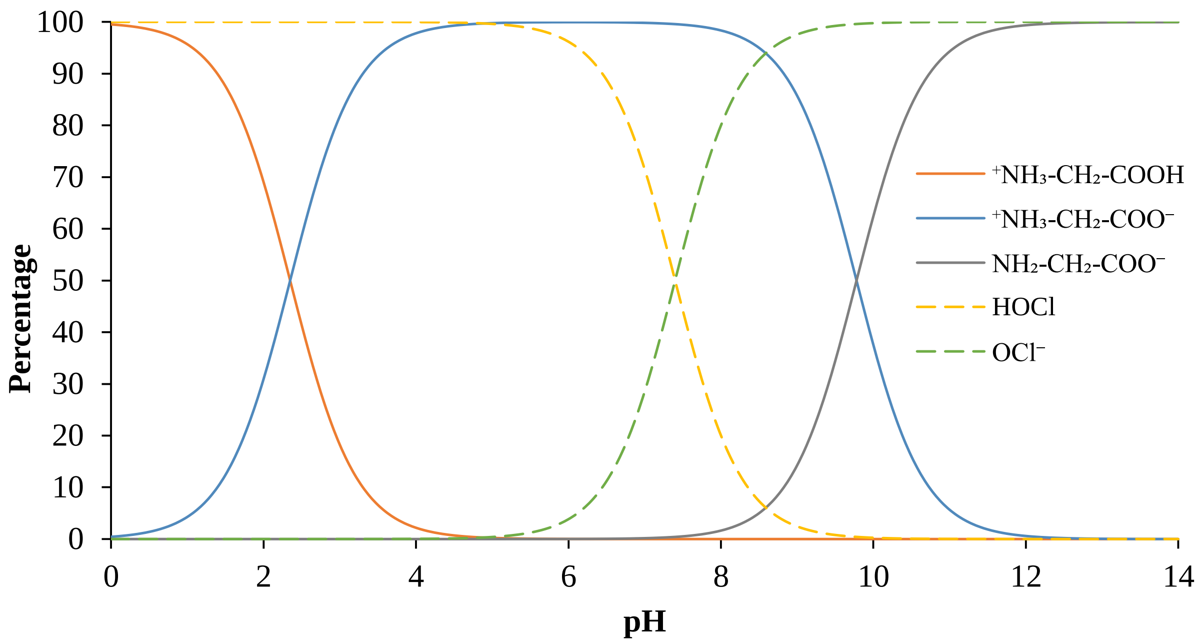

Figure S 1 Speciation of glycine and hypochlorous acid, p$K_{a(glycine\ carboxyl)}$ *= 2.35,* p$K_{a(glycine\ amino)}$ *= 9.78,* p$K_{a(HOCl)}$ *= 7.4* (1)

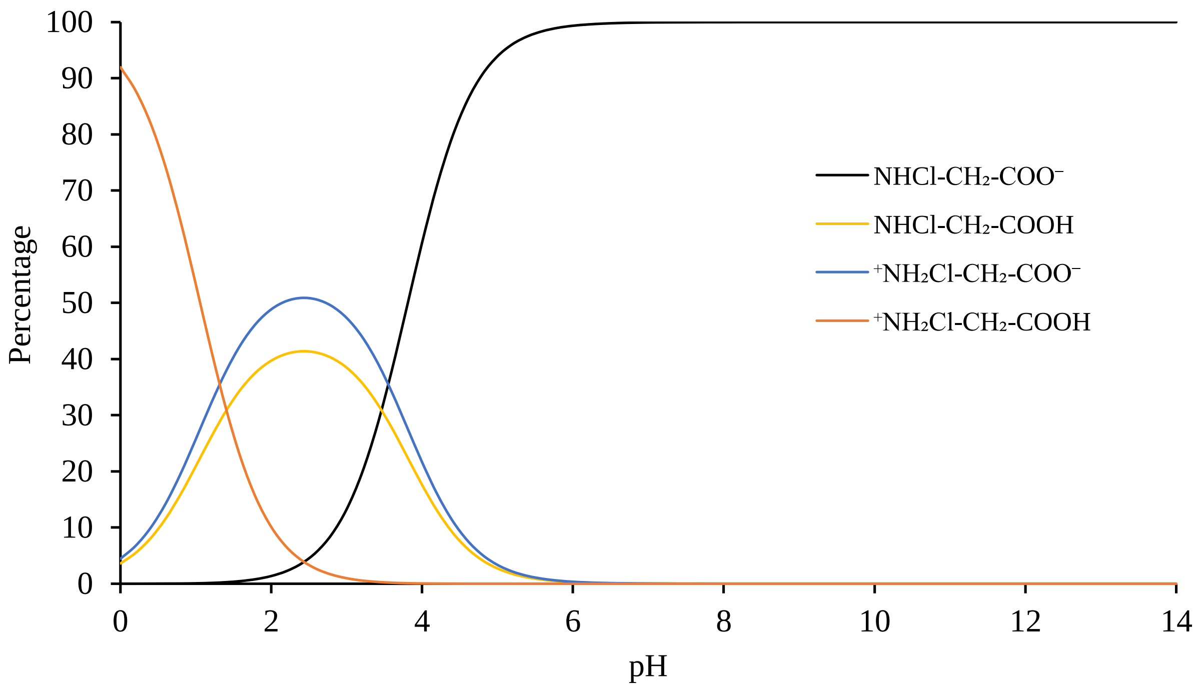

Figure S 2 Speciation of N-chloroglycine calculated by calculator Plugins MarvinSketch 19.3.0, 2019, ChemAxon (http://www.chemaxon.com) showing a pKa of 1.06 for carboxyl and 3.81 for amino group

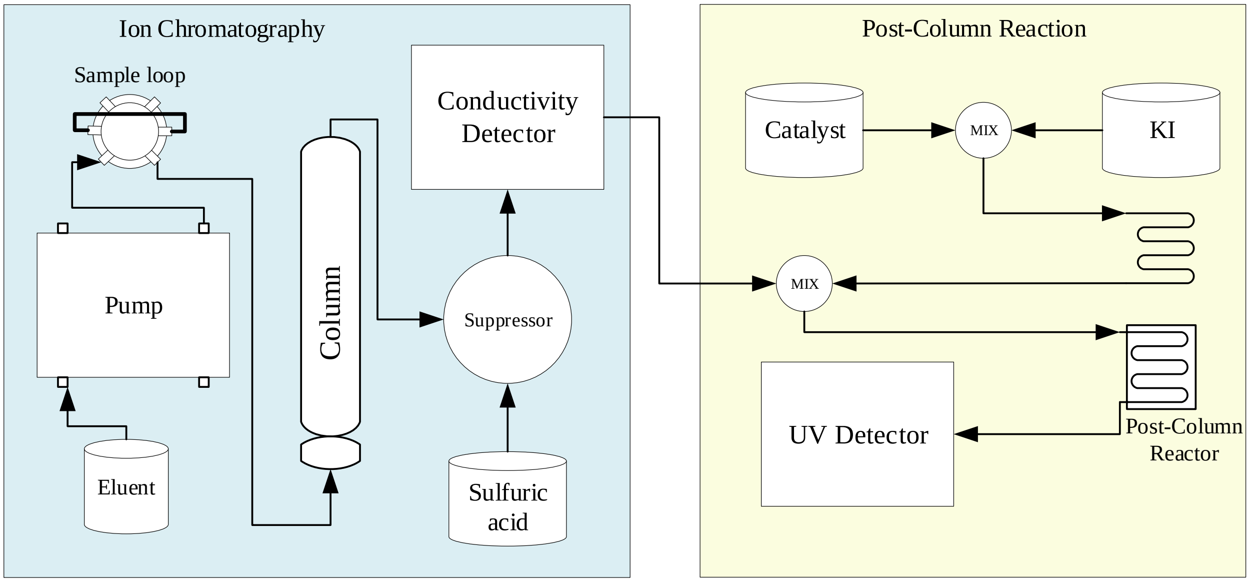

Figure S3 Schematic view of the IC and PCR system. Eluent: 1.6 mmol L^{-1} sodium carbonate, flowrate of 0.8 mL min-1, PCR: [KI] = 270 mmol L^{-1}, [ammonium molybdate tetrahydrate] = 50 µmol L^{-1}, [sulfuric acid] = 100 mmol L^{-1}, KI was added separately, flowrate of PCR reagents 0.2 mL min-1, wavelength of UV-detection: 352 nm, injection volume 300 µL

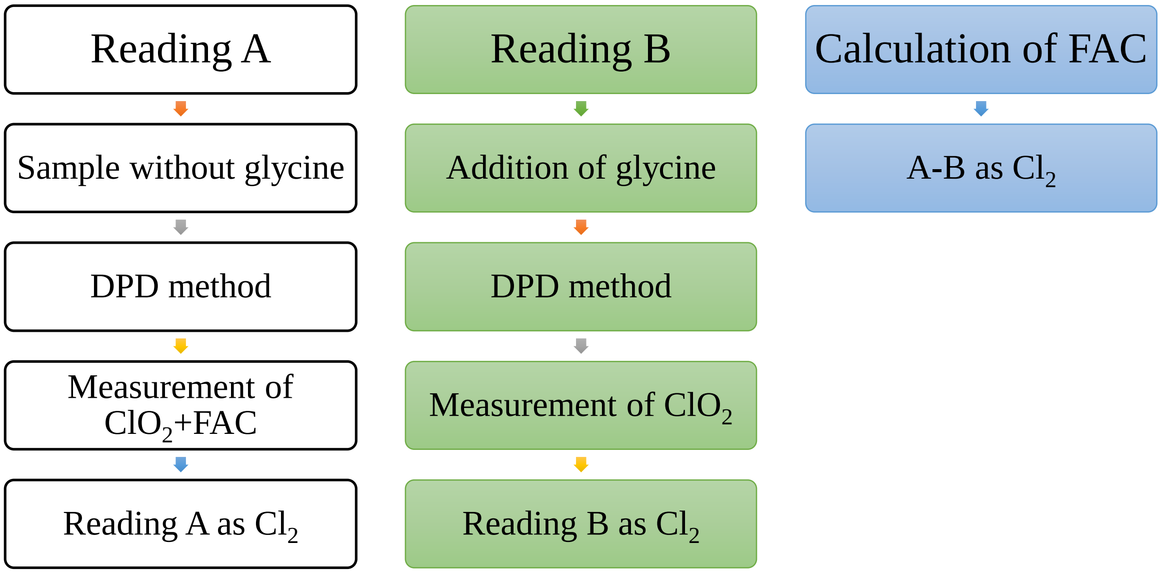

Figure S4 A graphical representation of DPD procedure for measurement of FAC in the presence of ClO_2

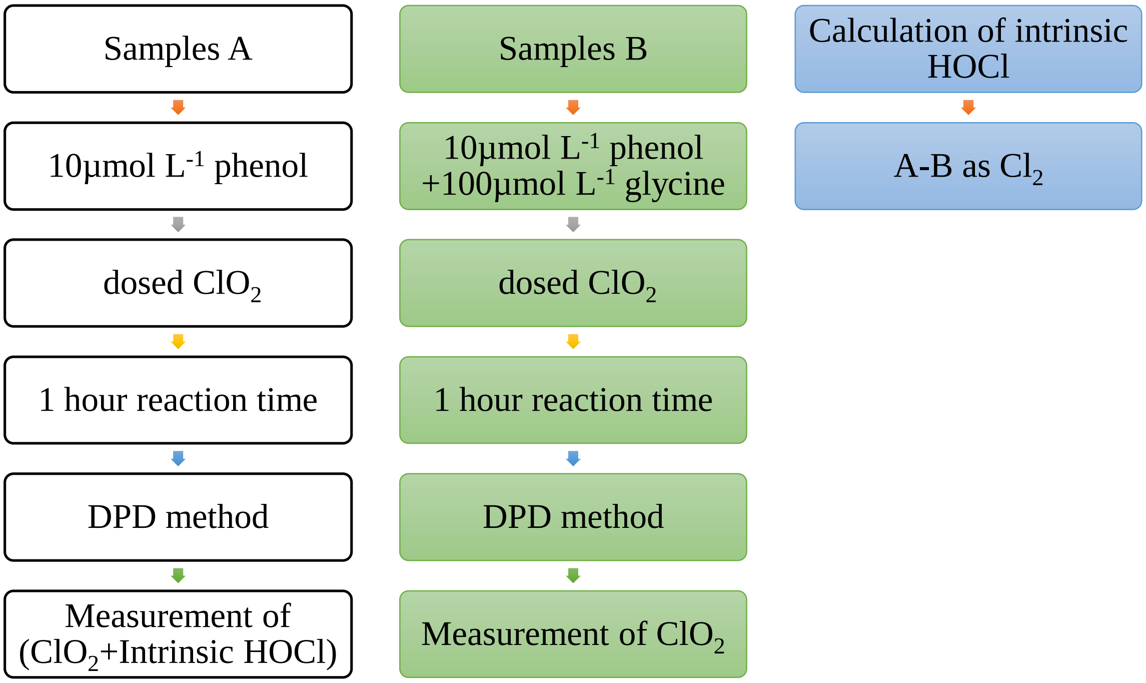

Figure S5 A graphical representation of DPD procedure for measurement of intrinsic HOCl formed in the reaction of ClO_2

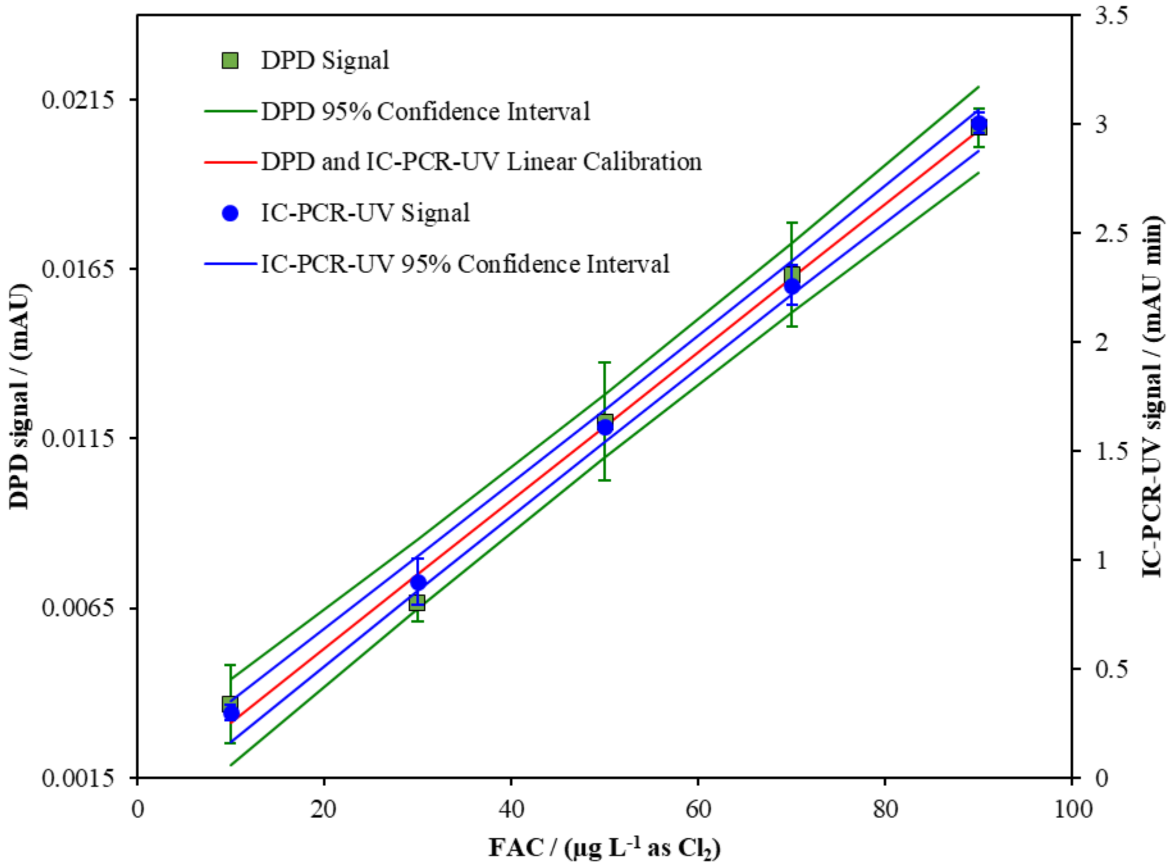

Figure S 6 Calibration and 95% confidence intervals for DPD and IC-PCR-UV methods. Error bars show the standard deviation of triplicate measurements. (FAC=added HOCl, expressed as Cl_2 equivalents)

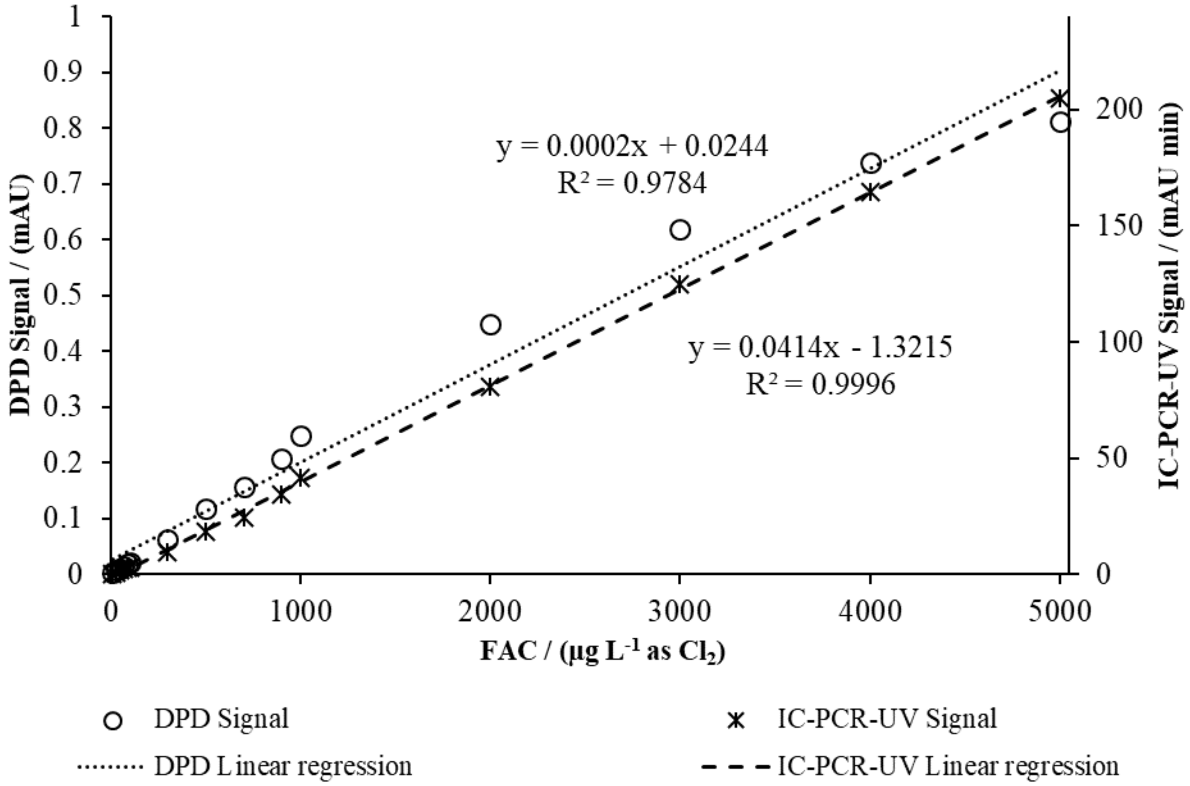

Figure S7 Calibration of FAC using DPD and IC-PCR-UV method. Different concentrations of FAC in ultrapure water are measured with DPD and N-chloroglycine methods.

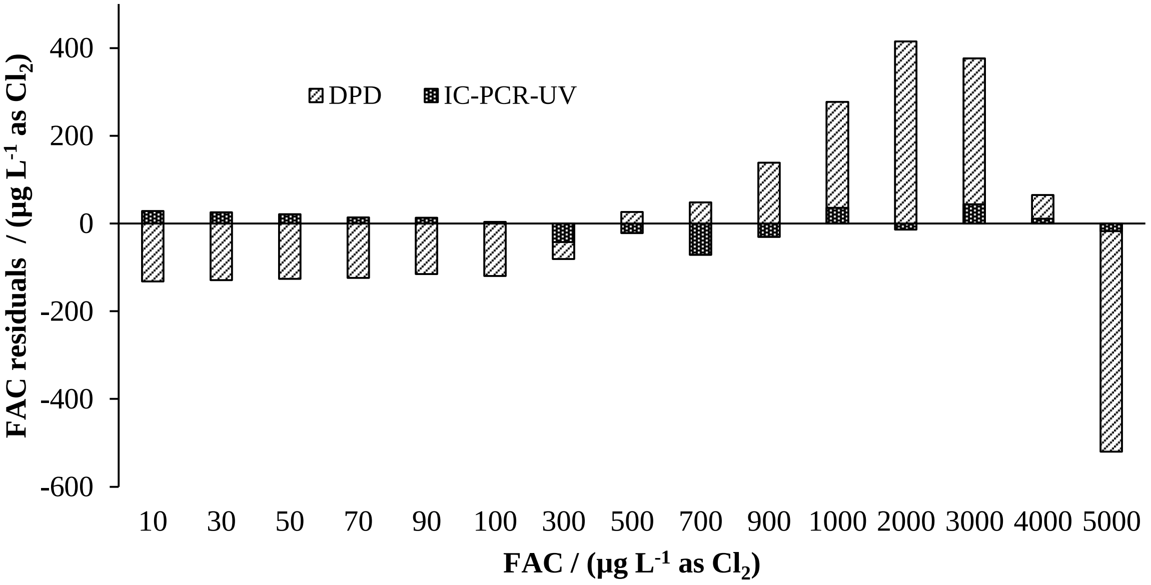

Figure S 8 Residuals of linear regression for DPD and IC-PCR-UV methods. (FAC = added HOCl, expressed as Cl_2 equivalents)

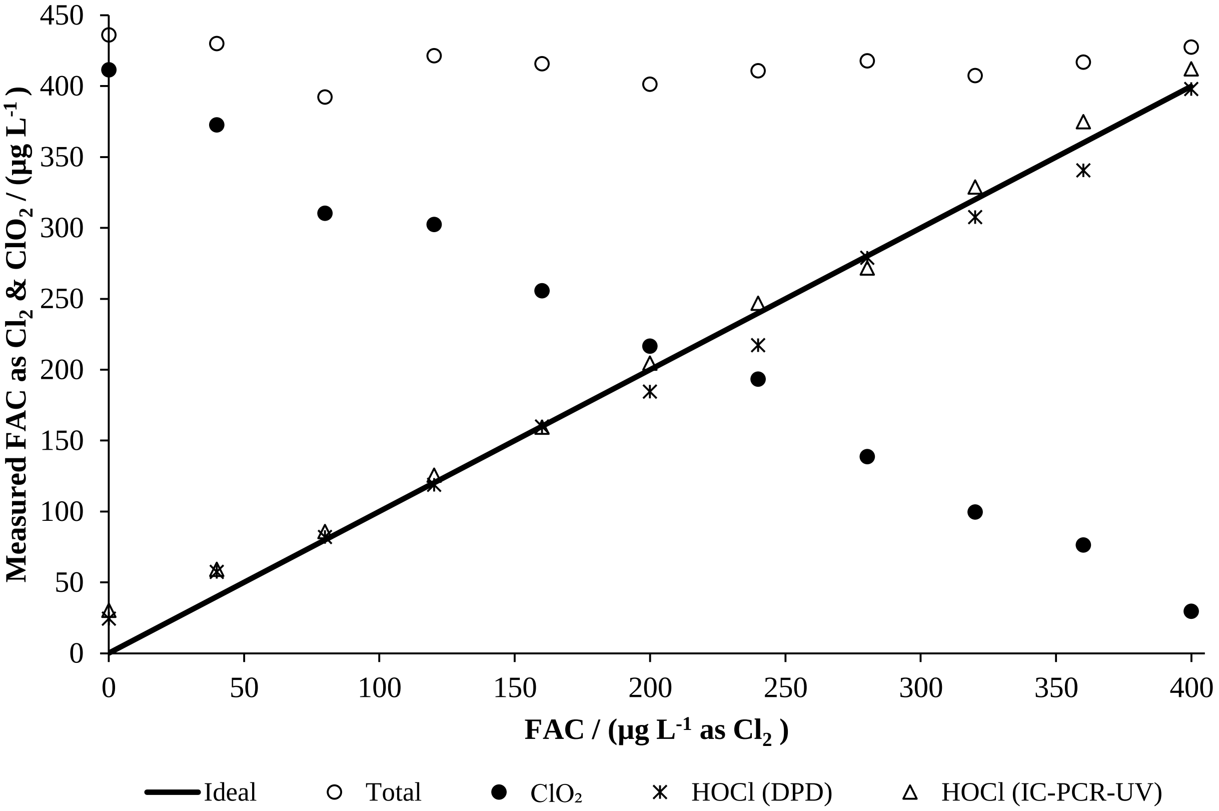

Figure S 9 Performance of DPD and N-chloroglycine method (IC-PCR-UV) for FAC measurement in the presence of ClO_2. To measure FAC by DPD method, "scavenged" samples (ClO_2) are subtracted from "not scavenged" samples (ClO_2 + HOCl). Different concentrations of FAC ranging from 0 to 400 µg L-1 are mixed with different ClO_2 concentration with 400 µg L-1 being the sum of FAC and ClO_2 (FAC = added HOCl, expressed as Cl_2 equivalents)

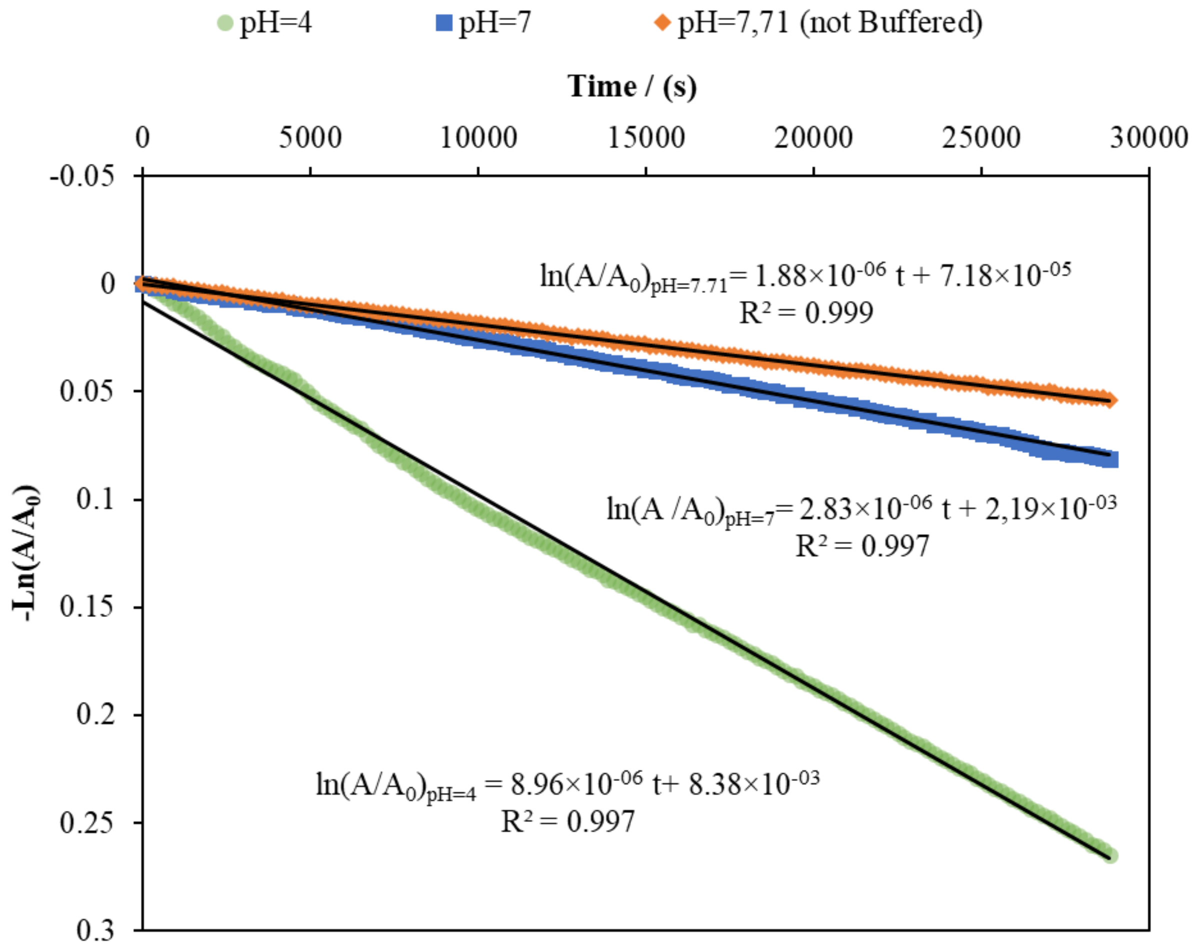

Figure S10 First order decomposition of N-chloroglycine at different pH values, [N-chloroglycine]$_0$ = 100 µmol L^{-1}, [phosphate buffer] = 5 mmol L^{-1}

Table S1 Anions of the water matrix measured alongside FAC in spiked tap water samples using N-chloroglycine method (IC-CD); Water sample taken at the University of Duisburg-Essen on August 24, 2018 with a pH of 7.80.

**Anion**	**Fluoride / (µg L^{-1})**	**Chloride / (mg L^{-1})**	**Bromide / (µg L^{-1})**	**Nitrate / (mg L^{-1})**	**Sulfate / (mg L^{-1})**
**Concentration**	133	63.3	114	4.69	33.0
**Confidence interval**	± 2	± 0.2	± 4	± 0.02	± 0.3
**Precision**	0.985	0.996	0.966	0.996	0.99

Text S1: Measurement of monochloramine by modified system

Due to the absence of anionic species for monochloramine and the presence of the conjugate acid to some extent (pK_a = 1.44 (2), Figure S11), monochloramine will not pass the ion suppressor. To selectively determine monochloramine in water samples, ion suppressor and conductivity detector can be bypassed. By using the PCR-UV detection, a separation-based quantification can be performed with this setup. This can selectively determine monochloramine and other ions that are capable of oxidizing iodide (e.g., chlorite, chlorate). The result from such set up is shown in Figure S12 and Figure S13. Due to the fact that this system cannot measure most conservative anions and needs higher skill levels to operate compared to cheaper methods already introduced to determine monochloramine, everyday use of such a system is not endorsed. However, it can be used to validate the performance of other methods for chloramine determination.

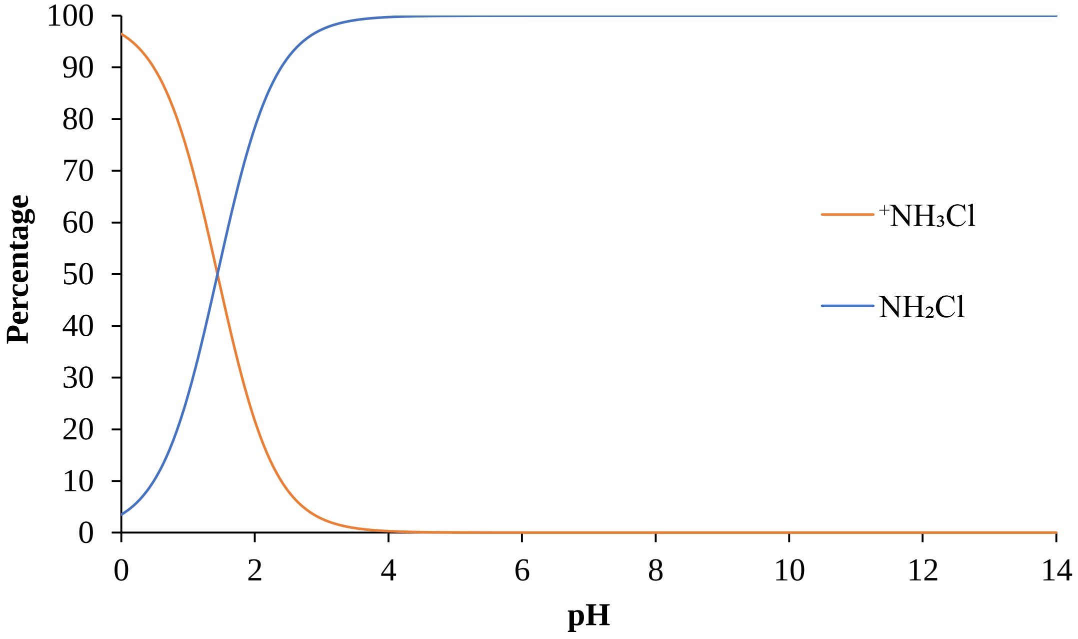

Figure S11 Speciation of monochloramine, pK_a *= 1.44* (2)

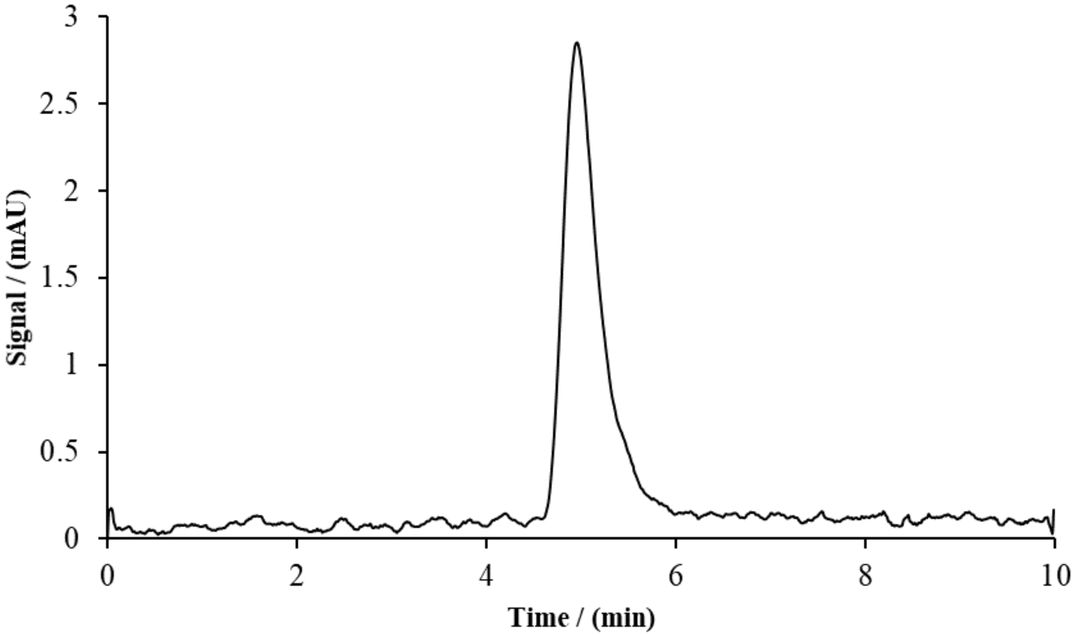

Figure S12 Chromatogram for separation of 5µmol L^{-1} monochloramine in IC-PCR-UV: separation column A Supp 4; Eluent 1.6 mmol L^{-1} Na_2CO_3+0.1 mmol L^{-1} $NaHCO_3$; Flowrate 1 mL min^{-1}; Sample loop 20µL

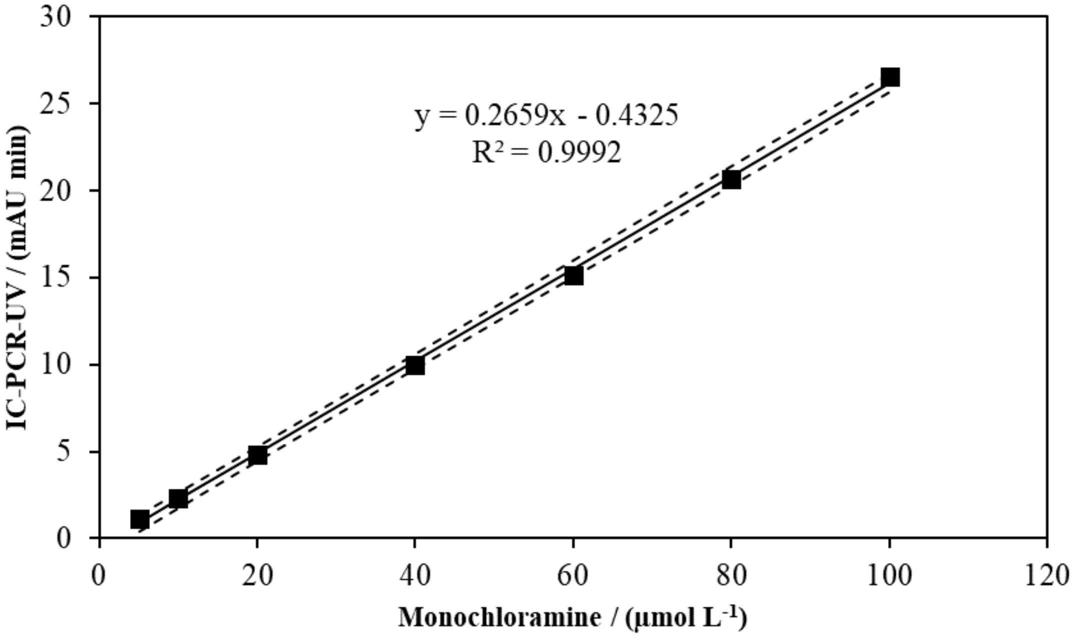

Figure S13 Calibration of monochloramine determined by IC-PCR-UV; separation column A Supp 4; Eluent 1.6 mmol L^{-1} Na_2CO_3+0.1 mmol L^{-1} $NaHCO_3$; Flowrate 1 mL min^{-1}; Sample loop 20µL

Text S2: *N*-chloroglycine decomposition

As all chloramines, *N*-chloroglycine is inherently unstable and decomposes according to Equations S1 and S2 (3–5).

$$ClHN-CH_2-COO^- \longrightarrow H_2C{=}NH + CO_2 + Cl^- \quad \textit{Equation S1}$$

$$H_2C{=}NH \xrightarrow{H_2O} CH_2O + NH_3 \quad \textit{Equation S2}$$

The most critical parameter affecting the stability of *N*-chloroglycine is pH (6,7). Therefore, the kinetics of *N*-chloroglycine decomposition was determined in different pH values for assessing the stability of samples to be measured by the *N*-chloroglycine method (Figure S10). The other factor of importance is the presence of hydrogen carbonate or any other naturally occurring proton donor, such as hydrogen phosphate (8). These compounds are acid catalysts and play a role in the disproportion reaction of chloramines.

The presence of the α-hydrogen in glycine can promote dehydrohalogenation. However, it seems that *N*-chloroglycine is relatively stable compared with other organic chloramines (9). A possible reason can be the absence of the alkane group in the α-carbon for glycine as the simplest amino acid. Organic chloramines such as *N*-chloroglycine can also undergo thermal decomposition (10), and the decomposition rate will decrease a lot at lower temperatures (6,7).

References

1. Lide DR. CRC Handbook of Chemistry and Physics. 90th ed. Boca Raton, Florida: CRC Press; 2010.

2. MARGERUM DW, GRAY ET, HUFFMAN RP. Chlorination and the Formation of N -Chloro Compounds in Water Treatment. In: ACS Symposium Series [Internet]. 1979. p. 278–91. Available from: http://pubs.acs.org/doi/book/10.1021/bk-1978-0082

3. Andrés J, Queralt JJ, Safont VS, Canle L M, Santaballa JA. Unimolecular Decomposition of the Anionic Form of N -Chloro-α-glycine. A Theoretical Study. J Phys Chem [Internet]. 1996 Jan;100(9):3561–8. Available from: http://pubs.acs.org/doi/abs/10.1021/jp9517209

4. Patai S. Amino, Nitrosco and Nitro Compounds and Their Derivatives: Vol. 1 (1982) [Internet]. Patai S, editor. Chichester, UK: John Wiley & Sons, Ltd.; 1982. 1438 p. Available from: http://doi.wiley.com/10.1002/9780470771662

5. Szabó M, Baranyai Z, Somsák L, Fábián I. Decomposition of N -Chloroglycine in Alkaline Aqueous Solution: Kinetics and Mechanism. Chem Res Toxicol [Internet]. 2015 Jun 15;28(6):1282–91. Available from: https://pubs.acs.org/doi/10.1021/acs.chemrestox.5b00084

6. Vikesland PJ, Ozekin K, Valentine RL. Monochloramine decay in model and distribution system waters. Water Res. 2001;35(7):1766–76.

7. Valentine RL, Jafvert CT, Leung SW. Evaluation of a chloramine decomposition model incorporating general acid catalysis. Water Res [Internet]. 1988 Sep;22(9):1147–53. Available from: https://linkinghub.elsevier.com/retrieve/pii/0043135488900103

8. Antelo JM, Arce F, Parajo M. Kinectic Study of the decomposition of N-chloramines. J Phys Org Chem. 1996;9(June 1995):447–54.

9. How ZT, Busetti F, Linge KL, Kristiana I, Joll CA, Charrois JWA. Analysis of free amino acids in natural waters by liquid chromatography-tandem mass spectrometry. J Chromatogr A [Internet]. 2014;1370:135–46. Available from: http://dx.doi.org/10.1016/j.chroma.2014.10.040

10. Ge H, Wallace GG, O'Halloran RAJ. Determination of trace amounts of chloramines by liquid chromatographic separation and amperometric detection. Anal Chim Acta [Internet]. 1990;237(C):149–53. Available from: http://www.scopus.com/inward/record.url?eid=2-s2.0-0025146596&partnerID=tZOtx3y1
